# Consulting with an embedded librarian: student perceptions on the value of required research meetings

**DOI:** 10.5195/jmla.2024.1793

**Published:** 2024-10-01

**Authors:** Susan R Franzen, Caitlin Stewart, Mallory Jallas, Joshua Newport

**Affiliations:** 1 srfranz@ilstu.edu, Associate Dean of Public Services and Organizational Development, Illinois State University, Normal, IL; 2 cjstew1@ilstu.edu, Teaching and Learning Librarian, Illinois State University, Normal, IL; 3 jallas2@uis.edu, Head of Information Literacy and Academic Outreach, University of Illinois at Springfield, Springfield, IL; 4 jcnewpo@ilstu.edu, Math and Science Librarian, Illinois State University, Normal, IL

**Keywords:** Embedded librarian, Qualitative Research, Consultations, Nursing Students, University Library

## Abstract

**Objective:**

This qualitative research project was undertaken to discover how students perceive the embedded librarian in their nursing class. The researchers determined how a required group research meeting was valued by students and whether that value warranted the necessary time and energy by an embedded librarian.

**Methods:**

Researchers conducted focus groups with twenty-three students from two different sections of the same nursing research methods undergraduate course. Students’ responses to a series of five questions were recorded within Zoom and supplemented by handwritten notes. Data was coded by hand and patterns that emerged from the five focus groups were analyzed.

**Results:**

Participants reported overall satisfaction with the embedded librarian and students felt they benefitted from the required research meeting with the librarian, which was part of the searching assignment rubric and closely tied to the assignment itself.

**Conclusion:**

Based on the data, a required research meeting with an embedded librarian, who is familiar with the course assignments, reinforces classroom instruction, point-of-need assistance with search strategies, and the opportunity to strengthen the relationship with the librarian for future research needs.

## INTRODUCTION

The benefits of embedded librarianship for university students broadly [1, 2, 3, 4, 5] and in nursing courses specifically [6, 7, 8] are well established in the literature. David Shumaker defines embedded librarianship as relationship-building that takes librarians out of the library and makes them part of a disciplinary team [1]. Others offer specific examples of embedded librarian activities such as point-of-need information instruction [9], physical presence in departments [10], virtual presence in library management systems [6], research consultations [11], and assessment of assignments [12]. Blake et. al. determined that due to embedded librarianship “Nursing students felt more confident in their literature searches, completion of assignments, and research. They also felt that librarian assistance led to an improvement in their grades” [7].

At Illinois State University, the practice of embedded librarianship for nursing students is an integral part of the curriculum. The required junior-level undergraduate nursing research methods class can be challenging for nursing students who have previously only taken hands-on skill-based courses; it is a high-impact course which uses embedded librarianship to improve student research skills. The research class has been a key course in the undergraduate nursing curriculum for many years. Prior to fall 2015, students were supported by one-shot library sessions. This ended when an embedded librarianship pedagogical model was integrated into the class.

The course's culmination is a collaborative research poster, and each assignment throughout the semester is designed as a component of this final project. The embedded nursing librarian attends classes regularly, provides three different information literacy sessions on finding, evaluating, and citing evidence, and plays a critical role in course content development, assignment development, instruction, and grading. Typically, the course is held in-person, however in spring 2021 the class met synchronously online. Paramount to the information fluency goals of the course was a required online, synchronous research meeting with the librarian to reinforce the material covered within the library instruction sessions.

Research consultations have been a staple of academic library services for years. Consultations can occur at a reference desk, in a consultation space, and within a virtual environment. Studies have shown that students feel more comfortable meeting with librarians in private spaces for consultations rather than in public spaces [13], and they prefer online sign-up for consultations as opposed to reaching out directly to a librarian [14, 15]. The embedded librarian used these best practice techniques when scheduling research meetings with the nursing students to make them more comfortable and to give them autonomy to find meeting times that work best in their schedules.

Student groups had to meet with the librarian in the first half of the semester to begin research for their collaborative poster. The research meeting has been a required component of the course since spring 2016 because voluntary research workshops offered by the librarian in fall 2015 had very low attendance and proved ineffectual in reinforcing library content presented in class. Four to five students met with the librarian for half an hour. The embedded nursing librarian held mandatory research meetings to assist students with the group search strategy assignment. As the creator of this assignment, the librarian was well-versed in the required components. Students also discussed initial articles they found with the librarian as well as sharing their PICOT questions for a discussion of possible search terms. The librarian graded the final submissions of the group search strategy assignment with the rubric she created (Appendix A).

Research consultations are often part of embedded librarianship within learning management systems and face-to-face courses. Librarians embedded in course sites and classrooms are often familiar with the curriculum and required research projects. Kamada et. al. remarks that being embedded in a course “enables the librarian to provide customized support for users’ complex information seeking activities” and gives librarians an advantage in assisting students with their literature searches and assessment of search results [10]. Research consultations empower students to apply what they learned in library instruction class sessions by providing a structured opportunity to organize information and make notes on search strategies and advice from the librarian [16]. Rogers … Carrier found students from various subject backgrounds value consultations to receive individualized attention from an expert [13]. Students who meet in consultation with a librarian report higher confidence in their research abilities because of increased knowledge of the research process and a stronger relationship with the librarian [17].

Within nursing and health sciences-related embedded librarianship literature, consultations are often mentioned in passing as one of many interventions [18, 19]. There is evidence in the literature that voluntary one-on-one consultations held by librarians are beneficial to students [11]. However, grade-dependent group consultations as a component of a research assignment are not described in the research literature. A required consultation with the nursing librarian aligns with the Kamada et al. assertion that ultimately, “…the embedded librarian model… aims to assist [pharmacy students] in proactive ways rather than waiting for users to seek assistance” [10]. There is evidence in the literature that required consultations can be more powerful and impactful as an extension of a library instruction session [20].

The effort and labor involved in coordinating and meeting with every student group is considerable; this led the embedded librarian to investigate the following questions: what were student attitudes towards the required intervention, how do undergraduate students perceive the value of a research meeting with an embedded librarian, and how does this value assist an embedded librarian to justify the time and effort required to build in research meetings in a course?

## METHODOLOGY

Applied thematic analysis was used to determine undergraduate nursing students’ perceptions of a required research meeting with the embedded librarian. Previous research at Illinois State University found from initial analysis of collected information fluency outcomes based on pre- and post-test data that “interactions with the embedded librarian had a positive impact on students’ skills” [12]. Due to the quantitative nature of the previous findings, a qualitative study was necessary to better understand the student perspective on the impact of recognized outcomes. Focus groups allowed for a rich qualitative analysis of the students’ experiences. Current students of the nursing research methods course were recruited as participants by their course instructor. A $50 gift card as an incentive for participation was made possible through a university research grant which helped to recruit participants. Approval for the study was obtained from Illinois State University's Institutional Review Board (IRB-2020-587).

There were 95 students in various sections of the nursing research methods course. In total, 23 of those students participated in a series of 4 focus groups with two researchers: a facilitator and a note-taker. Three of the focus groups had 6 student participants and one had 5. One additional student met with researchers and was interviewed individually due to limited scheduling flexibility. More than 24% of the sample frame were participants in the study. Focus group meetings occurred over video conference and were allotted 60 minutes, although sessions ended earlier if appropriate. To ensure open sharing, the embedded librarian was not part of the focus groups.

Focus groups consisted of an explanation, completion of unfinished consent forms, discussion of the questions, and concluding business with the incentives (Appendix B). Participants were encouraged to answer every question and add their thoughts to other participants’ answers. Some focus groups received follow-up questions and others did not. Follow-up questions were also responsive to participant answers and were not the same across focus groups when utilized. After the six planned questions were asked, the note-taker verbally summarized the responses to each question during the focus group. Participants were given the opportunity to say whether it was an accurate summary and to add additional comments/changes before the focus group concluded. Focus group notes also captured non-verbal communication such as nodding which would not be represented in a video conference transcript. These steps helped validate participants' focus group responses.

In addition to the notes, the sessions were also recorded and the closed caption feature in the video conference software was used to capture exact quotes from participants. The video conference transcript was cleaned and anonymized before analysis. The focus group transcripts and notes were reviewed by authors 2–4 (Caitlin Stewart, Mallory Jallas, and Joshua Newport) to analyze comments and to identify common themes using applied thematic analysis. Using Excel, authors 2–4 individually annotated the transcript for themes and developed a codebook based on their reading. Then, the themes and codebooks were compared, a shared codebook was created, and the transcripts were re-coded again using the same code book. It was then determined that the themes, which had been created to be relevant across all six questions, lacked specificity and failed to sufficiently capture the nuance within answers to specific questions, so a second analysis was undertaken to identify themes specific to each focus group question. To do this, authors 2–4 divided up the questions and their responses and created more granular themes which better reflected trends and anomalies in participant responses to the same question before collaboratively validating the findings. Finally, the main themes identified in this process were considered holistically and card sorted without classifying specific focus groups or question prompts to identify trends. The embedded librarian was only involved in the card sorting portion of the thematic analysis to mitigate possible bias. Quotations from participants were edited minimally for clarity and brevity.

## RESULTS

The themes that emerged during the focus groups indicated three main trends: a need for supplements to library instruction sessions, the research meeting serving as an important intervention point, and challenges faced with the research meeting.

### Need for More Than a Class Demonstration

This research methods course was not the first time most students worked with the embedded librarian. She also previously facilitated instruction with participants in their program orientation and skills courses. Some focus group participants remembered these past interactions with the nursing librarian, but the specifics of the library instruction were unclear. One individual recalled, “…I do remember now in [a previous course] her coming in and talking to us about the databases, but I didn't feel I retained that…” Other students did not remember that the embedded librarian had done instruction in other courses and commented that it would have been helpful to know her sooner: “I feel, though, it would have been helpful if I got to know her last semester, so that when I had to use the databases, I knew who specifically to contact because that was something I wasn't really sure how to do…” Despite attending previous courses, many of the students failed to retain the visit and the skills targeted from a class demonstration alone. This suggests that higher-impact strategies may be necessary for student learning.

Participants often needed individual or group repetition to be successful, beyond the class demonstration. As one participant shared, “…the meetings with [the embedded librarian] I think were extremely vital to make sure we were on the right path… just correcting anything that could have been misunderstood." The research meeting as a required check-in with the embedded librarian served as a point of contact for participants who had questions about expectations, skills, procedures, or more. Having multiple opportunities to learn or reinforce learning outcomes was important to many participants. Some participants expressed anxiety or shame over requiring repetition, but the nature of the research meeting normalized the asking for additional support and help. One participant shared with agreement from others that they still did not know how to search the database after it was covered in class, but the embedded librarian at the research meeting asked participants if they wanted to be shown again without the participant having to ask. One participant revealed, ”[The embedded librarian] was always down to just go ahead and reteach us this stuff, no matter how many times we're like ‘can you do that again’ she's fine with it." It is important to note that some participants felt the class demonstrations were sufficient but appreciated the embedded librarian being widely available, as one participant put it, “…once we were introduced to the online databases [during class] we didn't really have a hard time navigating them, but I would say the benefits definitely were her being there all the time in case we would need something…" While some participants needed additional support, others mentioned feeling confident searching after the class demonstrations.

### Research Meeting as Intervention Point

Participants named the research meeting as a key intervention to redirect their methods and yield a positive result. They generally found the meeting helpful - with some crediting it with their success on the assignment. This meeting is key for early identification of research challenges and timely intervention. It was especially helpful as a requirement for those who may have been unaware that they could improve their PICOT question and methodology. One participant shared, “I think going into it, we thought that all our stuff was really perfect and like we didn't really need to change anything. And then walking through it [with the embedded librarian] we realized… that maybe we didn't do this quite right.” This research meeting allowed an intervention point for students who would likely not have elected to confer with a librarian if it was not a requirement, and who would have otherwise not had the chance to course correct. For this to be successful, students must trust that the librarian is knowledgeable about the course requirements and practiced at the skills necessary.

Participants also identified that the meeting was a great opportunity to discuss approaches to research without feeling like a failure. As one participant stated with agreement from others, “It was just constructive criticism, and it didn't make us feel bad in any way. Usually, I'd be like, ‘Oh my gosh! Why did we do that?’ or like, ‘We're so dumb!’ or something, but… she made it a really nice environment…” Another participant shared, “When we met with her, she definitely commented on a lot of the things that we did, but it was… constructive criticism that helped us search a lot better and use our resources a lot better because we were a little confused on even how to use some of the databases and stuff.” They appreciated the embedded librarian's constructive feedback during project group meetings and felt it alleviated confusion.

Participants also reported that the meeting made their searching more efficient. Many students were searching and finding results but using strategies which meant they had to review a lot of results or peripherally relevant information. For example, a participant suggested, “the meeting definitely was helpful because I was just not searching in an efficient way, and I was sifting through all these articles that just weren't useful…” Another shared, “…when we went into the meeting, we kind of had no idea what we were doing with the whole project… [our approach] was very basic." Participants commented on the shift from using simple search strategies to doing something much more nuanced and targeted. Additionally, participants felt the embedded librarian was easily able to recognize where searches were going wrong and show them how to fix them. Participants described going to the embedded librarian with one idea for their project, and she would be able to tell students what was causing their problems and how to adapt, “because she just has familiarity with what we're looking up already.” This participant recognized that librarians who are accustomed to research often have an idea of what to expect, so if the search results are different from their expectation they know how to adjust and even that they need to adjust.

Participants appreciated the embedded librarian making suggestions to improve even when not encountering a problem. As one participant expressed, “Even if you didn't run into any problems, I feel like she would always give suggestions like ‘Oh, maybe try this if you want to further investigate’ or ‘Replace this with this,’ even if you were already coming up with terms.” Other participants agreed that the embedded librarian doing this was helpful. Participants were often happy with their search strategy if it was yielding results, allowing them to finish the assignment. The embedded librarian's suggestions to improve student searching pushed participants to think more critically about the process of research and acquiring deep skills that will be clinically effective. This showed the research process as one which is not entirely results-oriented.

Ultimately, participants identified many key skills in their research process as being strengthened or honed during the research meeting. These include explanations of database use; database limiters; Boolean logic; subject headings; critical evaluation; and PICOT questions. These various skills represent impact throughout stages of the research process. Example quotations for these skills are represented in Table 1.

**Table 1:** Participants’ perceptions of required research meetings on the research process

Research Skill	Participant Quote
PICOT Queastion	"So, our PICOT question, we had to basically make it more specific when we met with her. And I think if we didn't have that one-on-one meeting, we would have been going the wrong direction."
Database Use	"I would say probably learning how to use the different sources was the most helpful thing that I got from having the librarian in the class. [It] just, like, made me so much more comfortable and… just broadened what I was able to do when I was able to use more than one source, more than one database…"
Database Limiters	"I think I knew that there were all these limiters, but I didn't know either A) how to use them or B) what they meant… I could get to the list where a huge box of limiters [were] and I just didn't. I got overwhelmed and just kind of closed out of it before she walked us through it."
Boolean Logic	"She also walked us through how to use the AND, NOT, or OR… and I thought that was extremely helpful. She made it really clear like how useful those are and exactly how to use them."
Subject Headings	"Subject headings was something I didn't realize that databases had their own specific use, like specific words that are most useful in each individual database, so that was one of the new things that I learned. I had used limiters in the past, but that was something that I found useful."
Critical Evaluation	"I feel like I did just always trust… like, ‘oh, if it's in a database, that means it's a good article’, but just even knowing to question the quality of it, I feel like was such an important part. Even if we can't totally tell the quality of an article now, at least I know that's a question that I should be asking myself."

### Challenges with Research Meetings

Despite the overall effectiveness of the research meetings described by participants, some challenges were also disclosed. The scheduling and timing of the research meetings was chief among these. One participant explained that their project group had very limited shared availability outside of class, so the use of class time for the research meetings would have been ideal. They acknowledged that the embedded librarian was flexible regarding timing and coming up with a solution, but options were slim especially when project group members had childcare responsibilities and jobs. Several participants in the focus group also said that they wished the research meetings were longer as they ran out of time. Because the meetings were running over and scheduled back-to-back, participants recommend building in a transition time between group meetings. A project group which met with the embedded librarian earlier in the blocks of possible meeting times noted that it was challenging to have the meeting first because they did not have as much time to prepare or send materials to the embedded librarian ahead of time. As one participant summarized, “Once we were in the group, I do think she was very helpful and answered all of our questions and really… helped us narrow things down and figure out what we're doing, but definitely, the scheduling issue was probably the biggest challenge…” Given the benefits and challenges students brought to light, the required research meeting appears worthwhile.

## DISCUSSION

In the context of this nursing research class, students identified the project group meeting with the embedded librarian as critical to their research process (See Table 1). This meeting served as an intervention point that allowed students to ask questions, identify their assignment progress, address any discrepancies in their understanding, and get direct feedback from the embedded librarian. This meeting served as an extension and, in most cases, an expansion of the course instruction for students. Participants remarked that this meeting was a place to go more in-depth and engage critically with the skills they learned during their class time. In this research meeting, the embedded librarian models the research process by doing searches with the students and supports the project group's individual needs. Most critically, several participants noted this meeting as the turning point for their project where they were able to address fundamental issues with their work. Across all the focus groups, students consistently cited this intervention as important to their overall success in the course.

In addition to bolstering the student learning experience, the research meeting created individualized attention absent from the large online synchronous classroom. Incorporating this required meeting for student groups to facilitate their project in consultation with a librarian served as a vital connection point for the students. Noting an investment of time upfront in the assignment and building on the students’ perceptions of the embedded librarian as accessible aided in the process. The feedback from students in this course and the library literature confirm that opportunities for embedded librarians to meet the individual needs of students or their project groups are extremely impactful [10].

Group consultations are underrepresented in the literature which largely focuses on voluntary, individual consultations both when connected and unconnected from other embedded librarianship interventions [11, 13, 16, 17]. Interestingly, students noted in the focus group conversations that they were unaware that other students were struggling in similar ways and thought their project groups were the only ones with issues. The project group dynamics add a layer of complexity to this analysis by impacting student perceptions of their own research identity. In individual projects, students are isolated in their research skills and lack context for others. Through a group project, while that individual view of research competency remains, nuance is added by confronting the research skills of the group at large. The dynamic was not enough to normalize the research process for participants, instead, their perception shifted to assign their experiences and difficulties as being unique to their project group rather than common to all their class peers.

Research-based imposter syndrome emerged in certain participant responses. Specifically, transfer students in the focus groups shared that their perception was that their peers had already learned about research and the library, and they were disadvantaged or remedial in their approach. Among traditional students, there were comments about generally “trusting” database results too much and lacking the evaluative skills to select appropriate sources. There were also discrepancies in who felt equipped to successfully research based on the class demonstration alone and who needed repetition. This is made more complex due to some participants declaring that they felt prepared to research after the class demonstration alone but later identified the research meeting as helpful in completing the assignment. The initial confidence or anxiety of some participants after the class demonstration may have led to complicated project group dynamics.

The research meeting can help level the playing field and establish growth for all participants. These student perceptions highlight an opportunity for the embedded librarian to integrate and share context for where students are in their research process and facilitate conversation in the consultations that allow for the surfacing of some of these feelings. While participants did not mention it directly, the nature of the group project meant that students often divided work so that group members completed areas that played to their research strengths rather than developing all skills uniformly. This could potentially lead to future gaps in students’ research skills. The question emerged: how do we simultaneously yield individual research growth and successful project group outcomes? Also, what is an embedded librarian's responsibility to proactively build student researchers’ self-efficacy and push back against imposter syndrome in the research classroom? These questions were raised from discussions with participants but currently lack clear answers.

The value of required research meetings is contextualized by the high-time commitment of embedded librarianship and the scalability of new components. It is important to critically evaluate and reflect on the embedded librarianship methodology so that the student perception of interventions and logistical realities are balanced [6, 7, 11]. This equilibrium is important to maintain so that content and interventions are not added to the detriment of student learning outcomes or librarian well-being. Considering these variables, a required research meeting aligned with a class assignment as part of a larger embedded librarian program has true value from the student perspective. However, the ability to implement such an intervention requires sufficient staffing and resources to be meaningfully implemented.

A future direction for this research could integrate perspectives from teaching faculty collaborating with an embedded librarian to explore the impact of required group research meetings. In addition to this expansion, quantitative and qualitative assessments on required group research consultations in other instructional settings could help bridge gaps in the literature about this practice within academic librarianship.

## LIMITATIONS

There are some limitations to this study which are important to note. First and foremost, there was a time delay between the research meetings and the focus groups. Unfortunately, this meant that participants sometimes vocalized difficulty remembering the specifics of interventions and details of the research meeting.

Because students were all recruited from two course sections and a shared degree program, students had pre- existing relationships with other focus group participants. Some revealed that they were group members on the collaborative research poster. This could impact how students represented their experience, including confidence when researching and self-perception of skills and growth. Additionally, because of the small sample size and focused environment, results only demonstrate that these students in this context perceived value from the required research meeting.

Finally, the initial research question was focused on the student perception of embedded librarian practices more generally. However, the focus of the research project evolved to highlight the required research consultation due to its prevalence in participant responses. Data on other elements of the embedded librarian practices was collected but is not represented in the scope of this article. On occasion, participant comments did not clearly connect to a specific embedded librarian intervention but made more general statements. In those cases, conversational context was used to ascribe meaning.

While the questions were designed to allow participants to guide the conversation, in some cases that flexibility led to students incompletely responding to questions. Recommendations for future research would suggest critically evaluating the scope of questions. For example, in most groups focus group participants initially responded to the first question, “What benefits and/or challenges did you experience interacting with the librarian?” only with benefits. The facilitator began following up on this question specifically asking about challenges. This was important given the need to critically evaluate student perceptions, both positive and negative.

## CONCLUSION

The research meeting reinforces research skills from class demonstrations, provides point-of-need research support for the assignment, and nurtures relationships between librarian and student for the future. Given the need to balance high-impact embedded librarianship with scalability, reflecting on current practices with the lens of student perceptions is vital. This model of required group research consultation merits further exploration in the embedded librarianship literature from varied library settings. This assessment can inform local practice and leverage existing instructional relationships to constantly improve student learning outcomes in ways that are more nuanced than simply adding work or new strategies. Adding new instructional elements without assessing the impact of existing approaches can lead to burnout and can miss vital connections to student learning outcomes. The authors found that participants assigned a high value to the required group research meetings. However, for successful implementation, it also requires a librarian or librarians who highly value the principles of embedded librarianship and yield meaningful interactions with students.

## Data Availability

Data associated with this article cannot be made publicly available because they contain personally identifiable information. Access to the data can be requested from the corresponding author and may be subject to IRB restrictions.

## SUPPLEMENTAL FILES

**Appendix A:** Rubric

**Appendix B:** Focus Group Questions
