# Supplementary material for: Consulting with an embedded librarian: student perceptions on the value of required research meetings
Source: J Med Libr Assoc. 2024 Oct 7;112(4):324–31. doi: 10.5195/jmla.2024.1793 (PMC11486084; doi:10.5195/jmla.2024.1793)
Supplement: Supplementary file 1 — Appendix A: Rubric [file jmla-112-4-324-s01.docx]

# Appendix A

**Assignment Rubric**

| **CRITERIA** | **SATISFACTORY**  **4-5 points** | **DEVELOPING**  **2-3 points** | **UNSATISFACTORY**  **0-1 point** |
| --- | --- | --- | --- |
| PICO question and group member names included; Meeting with librarian complete | PICO question clearly stated; Group name and members’ names included; Meeting with librarian with completed Search Strategy Organizer submitted. | PICO questions unclear. Group or individual names not included. Meeting with librarian but Search Strategy Organizer incomplete or missing. | Omits/incomplete information. No meeting with librarian. |
| Clear record of searches conducted listed in chart | Clear and correct keywords, subject headings, limiters, and results included for all four databases. | Unclear or incorrect keywords, subject headings, limiters, and/or results for all four databases. | Information missing/minimal content; Fewer than four databases used |
| Narrative clearly explains CINAHL and PUBMED searches | Specific examples and clear explanation of how searches were conducted and revised; Strong understanding of strategies for finding best evidence. | General examples and explanation of how searches were conducted and revised; Basic understanding of strategies for finding evidence demonstrated. | Omitted information or minimal content about searches; No explanation of revisions; No indication of understanding search strategies. |
| Narrative clearly explains JBI and TRIP searches as well as number of articles selected | Specific examples and clear explanation of how searches were conducted and revised; Strong understanding of strategies for finding best evidence demonstrated; Number of articles selected clearly explained. | General examples and explanation of how searches were conducted and revised; Basic understanding of strategies for finding evidence demonstrated; Number of articles generally explained. | Omitted information or minimal content on searches; No explanation of revisions; No indication of understanding search strategies; Number of articles not listed. |

| Zotero folder | Folder shared correctly with group members, instructor, librarian and TA; All article citations within Zotero include PDF. | Folder not shared with all individuals; Some PDFs missing from article citations within Zotero. | No Zotero folder created and/or shared |
| --- | --- | --- | --- |
| Reference List | Reference list of 10-16 articles; uses APA format correctly throughout | Incomplete reference list and/or 5-10 APA errors | Incomplete reference list and more than ten APA errors |
