# Supplementary material for: Consulting with an embedded librarian: student perceptions on the value of required research meetings
Source: J Med Libr Assoc. 2024 Oct 7;112(4):324–31. doi: 10.5195/jmla.2024.1793 (PMC11486084; doi:10.5195/jmla.2024.1793)
Supplement: Supplementary file 2 — Appendix B: Focus Group Questions [file jmla-112-4-324-s02.docx]

Appendix B

Focus Group Questions:

1. What benefits and/or challenges did you experience interacting with the librarian?
2. How do you feel working with the nursing librarian impacted your ability to find literature in the databases?
3. What impact did the librarian have on your confidence in evaluating the literature you found in the databases?
4. How beneficial were your interactions with the librarian, specifically the group meeting, in preparing you to successfully complete assignment #3 or other assignments in the course?
5. All things considered, describe the one factor that is most significant about your success in NUR 336 in relation to interacting with a librarian throughout the course?
